# Chinese Cabbage Powder and Clove Extract as Natural Alternatives to Synthetic Nitrite and Ascorbate in Clean-Label Pork Sausages

**DOI:** 10.3390/foods14193316

**Published:** 2025-09-24

**Authors:** Jibin Park, Su Min Bae, Yeongmi Yoo, Minhyeong Kim, Jong Youn Jeong

**Affiliations:** 1 Department of Food Science & Biotechnology, Kyungsung University, Busan 48434, Republic of Korea; ppasd444@naver.com (J.P.); smbae219@gmail.com (S.M.B.); yooym9964@naver.com (Y.Y.); pleomax159@naver.com (M.K.); 2 Brain Busan 21 Plus Project Team, Kyungsung University, Busan 48434, Republic of Korea; 3 Food & Life Science Research Institute, Kyungsung University, Busan 48434, Republic of Korea

**Keywords:** clove extract powder, pre-converted Chinese cabbage powder, nitrite alternative, cured meat color, pork sausage, natural curing accelerator

## Abstract

The objective of this study was to evaluate the potential of clove extract powder (CEP) as a natural curing accelerator in pork sausages produced with pre-converted Chinese cabbage powder (PCCP) as a nitrite source. Sausages were prepared using a 3 × 2 × 2 factorial design with three levels of CEP (0, 500, and 1000 ppm), two sodium ascorbate levels (0 and 500 ppm), and two nitrite sources (synthetic sodium nitrite and PCCP). Increasing the level of CEP decreased pH, CIE L*, CIE a*, and residual nitrite, whereas CIE b*, cured meat pigment, total pigment, and curing efficiency were increased (*p* < 0.05). The inclusion of sodium ascorbate decreased (*p* < 0.05) residual nitrite levels while enhancing CIE b*, cured meat pigment, and curing efficiency. Compared with sodium nitrite, PCCP treatments retained higher residual nitrite (*p* < 0.05), although no significant differences (*p* ≥ 0.05) were observed for instrumental color, cured meat pigment, total pigment, or curing efficiency. These results demonstrate that CEP, when combined with PCCP, effectively promotes the development of cured meat color and enhances pigment stability, suggesting that this combination can serve as a promising alternative to synthetic nitrite and ascorbate in clean-label pork sausages.

## 1. Introduction

Nitrite is an essential curing agent that is commonly applied in meat processing because it imparts cured color, enhances flavor, and suppresses microbial growth, particularly that of *Clostridium botulinum* [1,2,3,4]. However, concerns regarding the potential health risks linked to synthetic nitrite, especially the generation of carcinogenic N-nitroso compounds, have led to increasing consumer demand for clean-label meat products that limit artificial additives [4,5,6,7,8]. Consequently, the meat industry is exploring natural alternatives to synthetic nitrites to maintain product quality and ensure food safety [9,10,11].

Among these alternatives, pre-converted vegetable powders have garnered considerable attention as natural nitrite sources owing to their ability to undergo microbial nitrate reduction [12,13,14]. Chinese cabbage (*Brassica rapa* ssp. *pekinensis*) is particularly promising in this context because it contains substantial amounts of nitrate, which can be converted to nitrite through fermentation [15,16]. Pre-converted Chinese cabbage powder (PCCP) has been shown to produce a cured meat color comparable to that of synthetic nitrite-treated products, while also offering additional advantages, such as enhanced color stability and oxidative resistance. Such properties are attributed to its rich content of polyphenols and organic acids, which act as natural antioxidants [15,16,17,18,19]. Clean-label meat products are generally understood as those made without synthetic additives, relying instead on familiar natural ingredients with minimal processing [11,14]. Although PCCP provides nitrite through microbial conversion of vegetable nitrate, its status as a clean-label ingredient remains under discussion, since regulatory definitions vary and consumers may not always perceive vegetable-derived nitrite as genuinely “natural” [20]. Previous research has also emphasized that while plant-based extracts are promoted as “green” solutions, further validation and safety assessments are required to align with clean-label standards [10,13].

In addition to nitrite sources, reducing agents are essential for accelerating the conversion of nitrite to nitric oxide, which subsequently reacts with myoglobin to form nitrosyl hemochrome, the main pigment responsible for the pink color of cured meat [3,20]. Conventionally, synthetic reducing agents such as sodium ascorbate and sodium erythorbate have served this role [2,3,20]. However, with growing interest in clean-label products, there is a substantial need to identify natural reducing agents that can achieve similar effects without synthetic additives [5,10,21,22]. Various studies have explored plant-based alternatives, such as green tea, rosemary, acerola, and dog rose, as potential replacements for synthetic ascorbates because of their high ascorbic acid and polyphenol content [12,23,24,25,26,27,28].

Clove (*Syzygium aromaticum*), a spice recognized for its distinctive aroma and strong antioxidant potential, is considered a promising natural reducing agent. It is rich in phenolic compounds such as eugenol, which exhibit radical scavenging and reducing activities [17,29]. Clove extracts have been found to have high phenolic content and potent antioxidant activity, supporting their potential to replace synthetic reducing agents while contributing to pigment stabilization and oxidative protection in cured meats [17,19,28]. Jin et al. [17] demonstrated that clove extract enhanced nitrite scavenging in emulsified pork sausages, whereas other reports highlighted benefits in reducing lipid oxidation and improving color stability [19]. Nevertheless, its combined application with pre-converted vegetable powders such as PCCP has not yet been fully investigated.

Therefore, the aim of this study was to evaluate the feasibility of replacing synthetic nitrite and ascorbate with PCCP and clove extract powder (CEP) in clean-label pork sausages. Specifically, the effects of different CEP concentrations, sodium ascorbate addition, and nitrite sources on the physicochemical properties of pork sausages were examined. These findings are expected to provide insights into the use of plant-based curing agents in the development of clean-label meat products.

## 2. Materials and Methods

### 2.1. Preparation of CEP

Whole cloves were purchased through an online retailer. After rinsing with running water to remove debris, excess surface moisture was removed using a salad spinner. The cleaned cloves were evenly spread on trays and dried in a hot-air oven at 40 °C for 12 h. The dried cloves were then ground using a hammer mill (LM 3100, PerkinElmer, Waltham, MA, USA) and sieved through a 600 μm mesh sieve. The resulting clove powder was vacuum-sealed in polyethylene bags with aluminum overwrap to prevent moisture uptake and light exposure and stored at –24 °C until use.

Clove powder was extracted by mixing the dried powder with 50% ethanol at a ratio of 1:50 (*w*/*v*). The suspension was heated and agitated in a shaking water bath at 80 °C for 30 min (100 rpm). After extraction, the mixture was cooled, transferred to centrifuge tubes, and centrifuged at 3400× *g* for 10 min at 4 °C (Combi R515, Hanil Science Industrial, Incheon, Republic of Korea). The resulting supernatant was filtered under vacuum using Whatman No. 1 filter paper, and ethanol was removed with a rotary vacuum evaporator (N-1000, Sunil Eyela, Seongnam, Republic of Korea). The concentrated extracts were frozen at –80 °C, freeze-dried at 5 Pa for 48 h (Lyoph-Pride, Ilshinbiobase, Dongducheon, Republic of Korea), and finally ground into a fine powder. The freeze-dried CEP was vacuum-packed and stored at –24 °C until analysis.

### 2.2. Preparation of PCCP

Chinese cabbage obtained locally (Busan, Republic of Korea) was washed, drained, and juiced using a commercial juice extractor. The juice was centrifuged at 3400× *g* for 10 min at 4 °C and then filtered through a 75 μm mesh sieve. The filtrate (approximate nitrate concentration: 2000 ppm, 53.41%) was inoculated with 0.1% starter culture (*Staphylococcus hominis* subsp.), along with 1% glucose, 0.06% oyster shell powder, and 45.43% water. Fermentation was carried out at 37 °C for 9 h with shaking at 100 rpm, as described by Kang [18]. After fermentation, the solution was pasteurized at 65 °C for 30 min, cooled in ice water, and maltodextrin was added to adjust the soluble solid content to 5%. The prepared mixture was frozen at –80 °C, freeze-dried, pulverized, vacuum-packed, and stored at –24 °C until further use.

### 2.3. Preparation of Ground Pork Sausages

Fresh pork ham muscles (*M. biceps femoris*, *M. semitendinosus*, *M. semimembranosus*) and back fat were obtained 24–48 h postmortem from a local processor. After trimming visible fat and connective tissues, the raw materials were cut into cubes (about 5 × 5 × 5 cm^3^). Pork and back fat were then separately ground using a meat grinder (TC-22 Elegant Plus, Tre Spade, Torino, Italy) equipped with a 3 mm plate.

Sausages were prepared according to a factorial design (3 × 2 × 2) that included three levels of CEP (0, 500, or 1000 ppm), two levels of sodium ascorbate (0 or 500 ppm), and two sources of nitrite (0.01% sodium nitrite or 0.44% PCCP) as shown in Table 1. The detailed formulations are provided in Supplementary Table S1. Based on the nitrite content of PCCP (22,878 ppm) determined in this study (see Section 3.2), an addition level of 0.44% was selected to provide an ingoing nitrite concentration of approximately 100 ppm, equivalent to 0.01% sodium nitrite in the final product. The sausage formulation consisted of 70% pork ham, 15% back fat, and 15% ice/water, with 2% NaCl and 1% sugar based on the total meat mixture weight. Initially, the ground pork was mixed with salt and either sodium nitrite or PCCP in a food mixer (5K5SS; Whirlpool, St. Joseph, MI, USA) at 120 rpm for 4 min. Subsequently, the back fat, sugar, sodium ascorbate, or CEP, along with the remaining ice/water, were added and mixed for an additional 4 min at the same speed. The mixture was then stuffed into 24 mm diameter cellulose casings (NOJAX^®^, Viskase^®^ Companies, Lombard, IL, USA) and cured at 3 °C for 24 h. The samples were cooked in a water bath at 90 °C until the core temperature reached 75 °C, monitored with a digital thermometer. Cooked samples were rapidly chilled in ice water for 20 min and stored at 3 °C until analysis.

### 2.4. Methods and Analyses

#### 2.4.1. Total Phenolic Content (TPC) and 2,2-Diphenyl-1-picrylhydrazyl (DPPH) Radical Scavenging Activity of CEP

The TPC of CEP was analyzed using the Folin–Ciocalteu method with minor modifications, following the procedure of Singleton et al. [30]. CEP was first diluted in distilled water to 1000 ppm. A 0.4 mL aliquot of the diluted solution was mixed with 2 mL of 10% Folin–Ciocalteu reagent and allowed to stand for 5 min, after which 1.6 mL of 7.5% Na_2_CO_3_ was added. The mixture was kept at room temperature for 120 min, and absorbance was subsequently recorded at 765 nm using a spectrophotometer (UV-1800, Shimadzu, Kyoto, Japan). Gallic acid (G7384, Sigma-Aldrich, St. Louis, MO, USA) served as the standard, and results were expressed as mg gallic acid equivalents (GAE) per gram of sample.

The DPPH radical scavenging activity was determined using a modified method outlined by Blois [31]. CEP was diluted to 1000 ppm with distilled water and 0.1 mL of this solution was combined with 3.9 mL of 60 µM DPPH reagent. The mixture was incubated for 30 min at room temperature, after which absorbance was measured at 517 nm using a spectrophotometer. Trolox (#238813, Sigma-Aldrich) was used as the calibration standard, and results were expressed as mM Trolox equivalents (TE) per gram of sample.

#### 2.4.2. Nitrate and Nitrite Ion Contents of PCCP

The concentrations of nitrate and nitrite ions in PCCP were analyzed using Merino’s zinc reduction method [32] with slight modifications. In brief, 5 g of PCCP was homogenized with 50 mL of pre-heated distilled water (80 °C), followed by clarification with Carrez solutions I and II, and centrifugation at 3400× *g* for 10 min. The resulting supernatant was filtered and diluted to a final volume of 100 mL with distilled water. For nitrite determination, aliquots of the extract were reacted with sulfanilamide and N-(1-naphthyl)ethylenediamine dihydrochloride (Griess reagents), and the absorbance was recorded at 540 nm using a UV–Vis spectrophotometer. For nitrate determination, an additional portion of the extract was mixed with 0.1 g zinc powder in ammonia buffer (pH 11.0) and gently agitated for 5 min to reduce nitrate to nitrite. The reduced solution was filtered, treated with the same Griess reagents, and absorbance was measured at 540 nm. The nitrate concentration was calculated as the difference between the total nitrite content after zinc reduction and the directly measured nitrite. Results were expressed as ppm of nitrate and nitrite ions.

#### 2.4.3. pH Measurement in Cured Pork Sausages

Five grams of sausage were blended with 25 mL of distilled water at 10,000 rpm for 1 min using a homogenizer (DI 25 basic, IKA-Werke, Staufen, Germany). The pH values of the homogenate were determined with a calibrated pH meter (Accumet^Ⓡ^ AB150; Thermo Fisher Scientific, Singapore).

#### 2.4.4. Cooking Loss in Cured Pork Sausages

Cooking loss was determined by comparing sample weights before and after cooking step used during manufacture and expressed as a percentage using the following equation: Cooking loss (%) = [(Weight before cooking − Weight after cooking)/Weight before cooking] × 100.

#### 2.4.5. Instrumental Color in Cured Pork Sausages

Color measurements were performed on freshly cut surfaces of the sausages using a chroma meter (CR-400; Konica Minolta, Osaka, Japan). The instrument was calibrated with a standard white tile (L* = +94.87, a* = −0.36, b* = +3.85) prior to use. Results were expressed in terms of CIE L* (lightness), a* (redness), and b* (yellowness). For each sausage, two inner surface points were selected on both sides, and four readings were recorded. The mean of these values was used for analysis.

#### 2.4.6. Residual Nitrite in Cured Pork Sausages

The residual nitrite content was determined according to the official AOAC method 973.31 [33]. Briefly, a 5 g sausage sample was homogenized with deionized water, heated to 80 °C for 2 h, cooled, and filtered to obtain a clarified extract. An aliquot (20 mL) of this extract was combined with 2.5 mL sulfanilamide solution and allowed to stand for 5 min. Subsequently, 2.5 mL of N-(1-naphthyl)ethylenediamine dihydrochloride solution was added, and the mixture was incubated for 15 min at room temperature. Absorbance was measured at 540 nm using a UV–Vis spectrophotometer. A standard curve was prepared using sodium nitrite (S2252, Sigma-Aldrich), and the results were expressed as ppm of residual nitrite.

#### 2.4.7. Cured Meat Pigment, Total Pigment, and Curing Efficiency in Cured Pork Sausages

The contents of cured meat pigment (nitrosyl hemochrome) and total pigment were determined according to the method of Hornsey [34]. For cured meat pigment analysis, 10 g of sausage was homogenized with 40 mL of acetone and 3 mL of distilled water, stored at 3 °C in the dark for 15 min, filtered, and the absorbance was read at 540 nm (A540). The cured meat pigment concentration was calculated as A540 × 290 (ppm, expressed on a meat weight basis).

For total pigment determination, 10 g of sausage was homogenized with 40 mL of acetone, 1 mL of HCl, and 2 mL of distilled water, and kept at 3 °C in the dark for 60 min, filtered, and the absorbance was measured at 640 nm (A640). The total pigment content was calculated as A640 × 680 (ppm, expressed on a meat weight basis).

Curing efficiency was expressed as the percentage ratio of cured meat pigment to total pigment [35].

### 2.5. Statistical Analysis

Each treatment was produced in three independent batches on separate days, and analyses were conducted in duplicate for samples from each batch. The experimental design followed a 3 × 2 × 2 factorial with CEP level (0, 500, or 1000 ppm), sodium ascorbate level (0 or 500 ppm), and nitrite source (0.01% sodium nitrite or 0.44% PCCP) as the main factors. Statistical analyses were performed using the GLIMMIX procedure in SAS software (version 9.4; SAS Institute Inc., Cary, NC, USA). Both the main and interaction effects were assessed, and significant differences among the treatment means were determined using the least squares means option *p* < 0.05.

## 3. Results

### 3.1. TPC and DPPH Radical Scavenging Activity of CEP

In this study, the TPC of CEP was found to be 302.30 mg GAE/g, indicating that CEP is rich in polyphenolic compounds. Ahmed et al. [29] reported that clove extract contains significant amounts of polyphenols such as gallic acid, catechol, protocatechuic acid, and quercetin, which are well known for their potent antioxidant activities. The DPPH radical scavenging activity of CEP was 1057 mM TE/g, which further demonstrates its strong antioxidant potential. This pronounced activity can be primarily attributed to the high phenolic content of CEP, especially eugenol, which has strong radical-scavenging capacity [36,37]. The potent antioxidant activity of CEP may promote the reduction of nitrite to nitric oxide during meat curing. Specifically, the phenolic compounds present in CEP exhibit strong reducing power, thereby facilitating nitrite conversion into nitric oxide. This reaction is essential for enhancing the formation of nitrosyl hemochrome, thereby improving the color and product stability of cured meat. Such effects have been indirectly supported by previous studies, which suggest that clove-derived polyphenols may accelerate nitrite reduction reactions and consequently lower residual nitrite levels during storage [17,38,39].

### 3.2. Nitrate and Nitrite Contents in PCCP

In this study, the nitrate and nitrite ion contents of PCCP were measured as 16,908 and 15,252 ppm, respectively, equivalent to 23,189 ppm of sodium nitrate and 22,878 ppm of sodium nitrite as their respective salt forms. This result demonstrates that the nitrate-reducing bacteria (*S. hominis* subsp.) employed during PCCP production efficiently converted nitrate into nitrite [18,40]. Previous studies revealed that natural nitrate sources and their subsequent conversion into nitrite by nitrate-reducing bacteria can provide curing effects, color stability, and microbial inhibition properties comparable to those of synthetic nitrites in meat products [38,39]. Thus, PCCP prepared in this study is expected to possess similar curing properties, being a promising natural curing agent for replacing synthetic nitrites in meat processing. Based on these results, the combination of PCCP, which has a high nitrite content, and CEP, which has a potent reducing capacity, can maximize the efficiency of curing reactions using natural reducing agents in meat products, thereby improving product quality and safety.

### 3.3. Effects of CEP, Sodium Ascorbate, and Nitrite Source on Physicochemical Properties of Naturally Cured Pork Sausages

#### 3.3.1. Overview of Main and Interaction Effects

The significance of the three main factors, CEP (C), sodium ascorbate (S), and nitrite source (N), together with their interaction effects on the physicochemical traits of naturally cured pork sausages, is summarized in Table 2. The dependent variables included pH, cooking loss, instrumental color values (CIE L*, a*, and b*), residual nitrite, nitrosyl hemochrome, total pigment content, and curing efficiency. Significant two-way interactions were observed for CIE b* and total pigment (C × S, *p* < 0.05), as well as for residual nitrite (S × N, *p* < 0.01). No significant three-way interactions were detected for any of the parameters evaluated (*p* ≥ 0.05). Accordingly, the results are presented in terms of the individual main effects and relevant two-way interactions for each dependent variable.

#### 3.3.2. pH and Cooking Loss

The effects of CEP, sodium ascorbate, and nitrite source on the pH and cooking loss of pork sausages are presented in Table 3. The pH significantly decreased with increasing levels of CEP (*p* < 0.05), with the 1000 ppm treatment affording the lowest value among all groups. However, no significant difference was observed between the treatments without CEP (0 ppm) and with 500 ppm CEP (*p* ≥ 0.05). In preliminary analyses, CEP had pH 4.05, which may explain the observed reduction in pH. This acidity is likely attributable to the polyphenolic constituents of clove, particularly the hydroxyl groups capable of releasing hydrogen ions [41]. Similar pH-reducing effects were also reported for red beet and purple sweet potato powders in pork sausages [42], where polyphenols and organic acids contributed to decreased pH values. The addition of sodium ascorbate (500 ppm) did not significantly alter the pH compared to treatments without ascorbate (*p* ≥ 0.05), which is consistent with previous findings for cured ground beef products [43]. Additionally, the nitrite source did not significantly affect the pH values, as no difference was observed between the synthetic nitrite- and PCCP-treated groups (*p* ≥ 0.05), which is in agreement with earlier findings using vegetable-derived nitrite sources [27,44].

Cooking loss significantly increased with higher concentrations of clove extract (*p* < 0.05), with the 1000 ppm CEP group showing the highest loss (6.77%; Table 3). No differences were found between the 0 and 500 ppm treatments (*p* ≥ 0.05), suggesting that high levels of acidic polyphenols may impair the water-holding capacity by affecting protein functionality. A similar reduction in yield was observed in sausages cured with white kimchi powder and natural antioxidants such as green tea and rosemary extracts [27]. Although no significant differences in overall pH were observed among treatments in this study, cooking losses still varied. This may be partly explained by the inherent buffering capacity of meat proteins [45], which helps stabilize the overall pH despite the addition of acidic compounds. The addition of sodium ascorbate had no significant effect on cooking loss (*p* ≥ 0.05). However, the nitrite source significantly influenced cooking loss (*p* < 0.05), with the PCCP-treated sausages showing lower values (5.16%) than those treated with synthetic nitrite (6.08%). This may be due to the dietary fibers naturally present in PCCP, which can enhance water-binding capacity, thus improving moisture retention [21]. In contrast, Choi et al. [23] reported greater cooking loss in products cured with vegetable-derived nitrite systems than with synthetic controls, whereas Jeong et al. [15] reported no differences in cooking yield between naturally and conventionally cured sausages. Overall, these findings suggest that cooking loss in naturally cured sausages is influenced not only by the concentration of plant extracts but also by the specific properties of the plant-based nitrite source and the inclusion or exclusion of phosphate or other formulation factors [27,45].

#### 3.3.3. Instrumental Color

The effects of CEP and PCCP on the CIE color values (L*, a*, and b*) of ground pork sausages were evaluated, and the results are presented in Table 3. As the level of CEP increased, the CIE L* values (lightness) showed a significant reduction (*p* < 0.05). Specifically, when the CEP level was increased from 0 to 500 ppm, the L* values decreased from 66.81 to 64.49, and further decreased to 63.47 at 1000 ppm (*p* < 0.05). These results suggest that the pigments inherent to CEP contributed to a darker cured meat appearance. Jin et al. [17] reported a similar decrease in the L* values of emulsified pork sausages upon the addition of 0.1% and 0.2% clove bud powder. Similarly, Liang et al. [46] reported that fried meatballs containing Chinese cabbage or cranberry powder had significantly lower L* values than those containing 60 mg/kg nitrite. However, the presence or absence of sodium ascorbate and the type of nitrite source did not affect the CIE L* values of the cured pork sausages (*p* ≥ 0.05). This result is consistent with the findings of Posthuma et al. [47], who reported that neither the nitrite source (sodium nitrite vs. celery juice powder) nor the presence of reducing compounds had a significant effect on the lightness (CIE L*) of cured meat model systems.

The CIE a* values (redness) decreased with increasing CEP level (*p* < 0.05; Table 3). Although the a* values remained similar between sausages treated with 0 and 500 ppm CEP, a further reduction was observed at the 1000 ppm level (a* 11.17), which was significantly lower than the value at 0 ppm (a* 11.35) (*p* < 0.05). However, despite the statistical significance, the difference in the CIE a* values between the two groups was very small. Zhang et al. [28] reported a similar dose-dependent reduction in the CIE a* values for clove extract concentrations ranging from 0.25% to 2% in Chinese-style sausages. Although the CIE a* values are commonly interpreted as indicators of nitrosyl hemochrome formation, previous studies have suggested that additional factors, such as the nature of plant-derived ingredients and the pigment composition of natural extracts, may also contribute to redness variations in meat products [17,48,49]. These mechanisms may collectively explain the decrease in redness observed at higher CEP levels. This hypothesis aligns with the findings of Jin and Kim [42], who reported that pigment-rich substitutes such as purple sweet potato and red beet affected redness and yellowness differently because of their distinct pigment compositions, including anthocyanins, betalains, flavonoids, and carotenoids. However, synthetic ascorbate-treated groups did not show significant differences in CIE a* values (*p* ≥ 0.05), although the strong reducing effect of ascorbate has been reported to enhance nitrosyl hemochrome formation [17,50]. In this study, the nitrite source had no effect (*p* ≥ 0.05) on the CIE a* values of pork sausages. Although nitrite is primarily responsible for developing the characteristic cured meat color through the formation of nitrosyl myoglobin and its heat-stable derivative, nitrosyl hemochrome, its impact on instrumental redness (CIE a*) can vary depending on the nitrite form and its interaction with other curing agents. However, in this study, the nitrite source (synthetic sodium nitrite vs. PCCP) did not result in significant differences in the CIE a* values of pork sausages (*p* ≥ 0.05), suggesting that the efficacy of pigment formation was comparable between the two nitrite systems. This finding is consistent with previous studies [44,49], where no significant differences in CIE a* values were observed among sausages prepared with radish powder despite variations in nitrate/nitrite content. Similarly, Magrinyà et al. [51] reported that the combined use of celery concentrate and starter culture in dry-cured sausages did not significantly influence the CIE a* values compared to traditionally cured sausages. Therefore, the lack of statistical difference in the CIE a* values observed in this study supports the potential of pre-converted vegetable powders as effective nitrite alternatives in clean-label meat products, provided that the conversion and reaction conditions are adequately controlled.

The CIE b* values (yellowness) of the cured pork sausages increased with higher CEP levels and with the inclusion of sodium ascorbate (*p* < 0.05; Table 3). Similar increases in the b* values have been observed in meat products formulated with plant-derived powders or extracts due to their inherent pigments [27,52,53]. In contrast, the nitrite source (synthetic sodium nitrite vs. PCCP) had no impact on the CIE b* values of the pork sausages (*p* ≥ 0.05). This result is consistent with previous findings suggesting that the effect of vegetable-derived nitrite sources on yellowness varies depending on the type and pigment profile of the plant material used [21,44,48]. For example, Horsch et al. [48] observed that increasing the concentration of celery concentrate led to increased yellowness in cooked ham products, which was attributed to the presence of pigment-containing plant-derived particulates. In contrast, Yoon [44] and Bae et al. [21] reported that sausages cured with radish or Chinese cabbage powder exhibited CIE b* values comparable to those cured with synthetic nitrite, suggesting that not all plant-derived nitrite sources significantly alter the yellowness of cured meat products.

#### 3.3.4. Residual Nitrite Content

The residual nitrite content of ground pork sausages was significantly influenced by CEP, sodium ascorbate, and the nitrite source (*p* < 0.01; Table 4). As the concentration of CEP increased, the residual nitrite produced a clear dose-dependent decline, from 36.36 ppm at 0 ppm CEP to 33.41 ppm at 1000 ppm CEP (*p* < 0.05). This agrees with earlier findings that polyphenol-rich plant extracts such as green tea, grape seed, and rosemary lower residual nitrite by facilitating its conversion to nitric oxide [54,55,56]. These results suggest that phenolic compounds in clove extract similarly act as natural reductants, contributing to nitrite depletion and the curing reaction in meat systems.

In conventional meat curing systems, synthetic reducing agents such as sodium ascorbate and erythorbate play the same role. In this study, the addition of sodium ascorbate (500 ppm) reduced the residual nitrite from 54.10 ppm to 15.42 ppm (*p* < 0.05; Table 4), in line with previous reports that ascorbate accelerates nitrite reduction to nitric oxide, thereby lowering residual nitrite while enhancing cured pigment formation [47,57,58].

The source of nitrite significantly affected the residual nitrite content. Sausages cured with PCCP retained higher residual nitrite (37.45 ppm) than those cured with synthetic nitrite (32.08 ppm) (*p* < 0.05). This difference may reflect nitrate-derived compounds that persist following microbial conversion and help stabilize nitrite, thereby slowing its complete depletion [59,60]. Such stabilization can be beneficial, maintaining a small nitrite reservoir that supports pigment stability and microbial safety [61], but it also underscores the dual nature of residual nitrite. In this study, the values remained within regulatory limits and were only slightly greater than those of the sodium nitrite controls. Notably, earlier research has shown that polyphenols and ascorbic acid can inhibit nitrosamine formation [25], suggesting that the combined use of PCCP and CEP may help mitigate toxicological risks. Rasmussen [60] also observed higher residual nitrite in systems with pre-converted celery powder compared to sodium nitrite, indicating that plant-derived compounds may interfere with nitric oxide formation or promote nitrite regeneration. Collectively, these results demonstrate that plant-based nitrite sources often retain more residual nitrite due to chemical and biological interactions; however, when paired with effective reductants, they can still achieve both technological and safety objectives in naturally cured meat products.

#### 3.3.5. Cured Meat Pigment, Total Pigment, and Curing Efficiency

Nitrosyl hemochrome is the primary cured pigment responsible for the characteristic pink-red color of cured meat products. It forms when nitric oxide, generated by nitrite reduction, binds to the heme group of myoglobin, producing a heat-stable pigment [2,3,62]. The stability and concentration of nitrosyl hemochrome are widely regarded as indicators of curing efficacy and the visual quality of processed meat.

Among the tested factors, the addition of CEP significantly increased the nitrosyl hemochrome in cured pork sausages (*p* < 0.05; Table 4). The 1000 ppm CEP treatment exhibited a higher concentration than the 0 and 500 ppm groups (*p* < 0.05), whereas latter two did not differ (*p* ≥ 0.05). Interestingly, this increase occurred despite the reduced CIE a* values in the 1000 ppm CEP group, suggesting that the darker coloration was not due to lower pigment formation but rather to the inherent color of clove extract. Thus, instrumental redness may not always reflect the nitrosyl pigment levels when highly pigmented plant extracts are present [63]. These findings are consistent with previous reports showing that polyphenol-rich plant extracts promote cured meat pigment formation. Gao et al. [55] demonstrated that theaflavins and tea polyphenols significantly increased nitrosyl hemochrome levels in cured sausages. Yoon et al. [27] observed that white kimchi powder combined with green tea or rosemary extract yielded nitrosyl hemochrome contents comparable to sausages cured with synthetic nitrite and ascorbate. Similarly, Wójciak et al. [64] found that blackcurrant leaf extract (150 mg/kg) enhanced nitrosyl pigment formation even at reduced nitrite levels, highlighting the reductive efficacy of phenolic compounds. In this study, sodium ascorbate (500 ppm) significantly enhanced nitrosyl hemochrome formation (*p* < 0.05), with ascorbate-treated sausages reaching a pigment concentration of 40.27 ppm, compared to 36.33 ppm in the ascorbate-free group (*p* < 0.05). This reflects the strong reducing ability of ascorbate, which accelerates nitric oxide release and stabilizes the nitric oxide–myoglobin complex [35]. Such results align with previous studies demonstrating that both synthetic (sodium ascorbate and erythorbate) and natural (acerola and cherry powder) reductants enhance nitrosyl hemochrome formation in clean-label curing systems [23,47]. In contrast, the nitrite source did not influence the nitrosyl hemochrome content (*p* ≥ 0.05). Samples cured with synthetic nitrite (38.02 ppm) and PCCP (38.59 ppm) yielded comparable values (*p* ≥ 0.05). Although the PCCP-treated sausages retained more residual nitrite, this did not lead to higher cured pigment formation or CIE a* values. This suggests that cured color development depends more on the efficiency of nitrite reduction and availability of reductants (ascorbate or plant polyphenols) than on the residual nitrite concentration. Jeong et al. [15] similarly reported that sausages cured with spinach powder showed higher residual nitrite contents but lower CIE a* values than those with other vegetable powders. Yoon [44] similarly observed no significant difference in nitrosyl hemochrome between sausages cured with synthetic nitrite (36.09 ppm) and radish powder (35.72 ppm). Collectively, these results indicate that pre-converted vegetable powders can act as effective nitrite alternatives, capable of generating cured meat pigment levels comparable to synthetic nitrite.

The total pigment reflects the concentration of heme-derived pigments and is crucial for the characteristic red-pink color of cured meat, which affects consumer acceptance [35]. In this study, CEP addition to cured pork sausages increased the total pigment content (*p* < 0.05; Table 4). Sausages treated with 1000 ppm CEP exhibited a significantly higher total pigment content than those treated with 0 and 500 ppm (*p* < 0.05), while no significant difference was observed between the 0 and 500 ppm groups (*p* ≥ 0.05). This trend closely paralleled that of nitrosyl hemochrome formation, suggesting that the enhancement in the total pigment levels may be primarily attributed to the increased formation of stable cured pigments. Several studies have also reported a positive correlation between the total pigment and nitrosyl hemochrome content in cured meat systems [21,49,65]. In addition to clove extract, various plant- and herb-derived ingredients have been shown to support or preserve the total pigment concentrations in naturally cured meat products. Yoon et al. [27] evaluated the effects of green tea and rosemary extract powders on sausages formulated with white kimchi powder and revealed that the total pigment levels were statistically similar to those of conventional controls prepared with sodium nitrite and ascorbate, regardless of the extract concentration. Similarly, Terns et al. [58] demonstrated that cherry powder maintained total pigment concentrations comparable to those of the nitrite-treated controls in indirectly cured ham systems. Conversely, the addition of sodium ascorbate (500 ppm) did not noticeably alter the total pigment levels (*p* ≥ 0.05). Sausages formulated with and without ascorbate exhibited statistically similar concentrations, indicating that the presence of ascorbate did not substantially alter the aggregate pigment profile. Similarly, the type of nitrite source did not influence the total pigment content (*p* ≥ 0.05); PCCP-cured sausages (49.05 ppm) and synthetic nitrite-cured samples (49.33 ppm) showed no statistical difference (*p* ≥ 0.05). These outcomes are consistent with those reported by Jeong et al. [15] and Gwak [49], who demonstrated that vegetable-derived nitrite sources, such as Chinese cabbage and radish powder, could effectively sustain total pigment levels equivalent to those achieved using synthetic nitrite.

Curing efficiency, expressed as the ratio of nitrosyl hemochrome to the total pigment, serves as a practical measure of curing effectiveness in meat products [35]. In the present study, ground pork sausages with CEP showed higher curing efficiency than those without it (*p* < 0.05; Table 4). Sausages without CEP (0 ppm) exhibited the lowest value (75.53%), whereas the addition of 500 ppm and 1000 ppm CEP increased curing efficiency to 78.30% and 79.95%, respectively (*p* < 0.05). These outcomes indicate that CEP promotes the conversion of nitrite into nitric oxide and the formation of stable cured pigments, a pattern also observed for other fruit- and herb-based ingredients used in meat processing [21,47,55]. This effect is likely associated with the antioxidant capacity of clove extract, which contains eugenol and abundant polyphenols [17]. Moreover, the curing efficiency was significantly influenced by the addition of sodium ascorbate (*p* < 0.05); the addition of 500 ppm synthetic ascorbate achieved the highest curing efficiency (81.86%), which was markedly higher than that of samples without ascorbate addition (73.99%) (*p* < 0.05). This result aligns with the well-documented role of ascorbate as a reducing compound that accelerates nitrite reduction and supports the generation of nitrosyl hemochromes, thereby improving cured color stability [2,3,14]. Serdaroğlu et al. [50] demonstrated that the co-application of pre-converted arugula and barberry extracts in heat-treated fermented sausages afforded a curing efficiency of 83.52%, indicating that plant-based nitrite sources coupled with antioxidant-rich extracts can effectively replace synthetic nitrite systems. Similarly, Posthuma et al. [47] reported that the use of cherry powder-derived ascorbic acid in a model meat system significantly increased the percentage of cured meat pigments to more than 72%, a value comparable to that obtained using conventional sodium erythorbate. These studies confirm that natural reductants can effectively enhance nitrosyl pigment formation. However, the nitrite source itself did not significantly affect curing efficiency (*p* ≥ 0.05). Sausages treated with sodium nitrite (77.09%) and PCCP (78.76%) exhibited similar curing efficiencies (*p* ≥ 0.05). This finding implies that when paired with suitable reducing agents or antioxidants, vegetable-based nitrite sources are as effective as synthetic nitrite in maintaining cured color and pigment stability [44,47,58]. While Magrinyà et al. [66] reported a lower curing efficiency in sausages cured with vegetable concentrate and *Staphylococcus carnosus*, Yoon [44] observed no significant differences between synthetic nitrite (80.74%) and radish powder with starter culture (79.49%), supporting the current findings. Therefore, these findings indicate that both clove extract and sodium ascorbate contribute to improved curing efficiency, and that the use of natural antioxidants provides a realistic strategy for producing clean-label cured meat products.

#### 3.3.6. Interaction Effects of CEP and Sodium Ascorbate on CIE b* and Total Pigment

Significant two-way interactions between CEP and sodium ascorbate were observed for the CIE b* and total pigment values (*p* < 0.05; Table 5). The CIE b* values of pork sausages increased significantly as the CEP concentration increased from 0 to 1000 ppm, regardless of the presence of sodium ascorbate (*p* < 0.05). This suggests that the yellow pigments derived from the clove extract exhibited a concentration-dependent effect. Zhang et al. [28] reported a similar trend in Chinese-style sausages containing clove extract, while other studies also showed that inherent pigments in plant-based powders can elevate the b* values [27,52,53]. At the 500 ppm CEP level, sodium ascorbate addition resulted in higher b* values than treatments without it, indicating a possible synergistic effect (*p* < 0.05). However, at 0 or 1000 ppm CEP, no significant differences were observed between ascorbate-treated and untreated groups (*p* ≥ 0.05), implying that sodium ascorbate plays a limited role in modifying yellowness.

A statistically significant reduction in the total pigment was observed in the 0 ppm CEP group with ascorbate compared to that without ascorbate (*p* < 0.05; Table 5); however, the magnitude of this difference was minimal. In contrast, sausage samples with and without ascorbate did not differ when 500 or 1000 ppm CEP was added (*p* ≥ 0.05), suggesting that CEP may contribute directly to total pigment formation. Moreover, regardless of ascorbate addition, the total pigment content increased consistently with higher levels of CEP (*p* < 0.05), supporting the pigment-stabilizing function of the phenolic compounds present in the clove extract. The effect of ascorbate on total pigment content may be less pronounced when clove extract is present in the formulation, possibly due to the dominant influence of polyphenolic compounds on pigment retention in naturally cured meat products. Similar pigment-stabilizing effects have been reported in other studies, with Yoon et al. [27] showing that white kimchi powder supplemented with green tea or rosemary extract powders achieved pigment contents comparable to those of sodium nitrite and ascorbate. Similarly, Gao et al. [55] demonstrated that tea polyphenols and theaflavins improve the stability of cured pigments in sausages, possibly by facilitating nitric oxide formation and protecting pigments from oxidative degradation.

#### 3.3.7. Interaction Effects of Sodium Ascorbate and Nitrite Source on Residual Nitrite

A significant two-way interaction between synthetic ascorbate and nitrite source affected the residual nitrite content in cured pork sausages (*p* < 0.01; Table 5). In the absence of ascorbate, sausages prepared with PCCP showed a higher residual nitrite content (57.71 ppm) than those with synthetic nitrite (50.49 ppm) (*p* < 0.05). Upon ascorbate addition (500 ppm), residual nitrite was significantly reduced to 17.18 ppm with PCCP and 13.67 ppm with synthetic nitrite (*p* < 0.05). These findings confirm a strong interaction effect, indicating that ascorbate consistently accelerated nitrite depletion across treatments. However, PCCP sausages still maintained higher residual nitrite than sodium nitrite counterparts, even in the presence of ascorbate (*p* < 0.05). This outcome may be associated with the nitrate and polyphenol constituents of PCCP, which could stabilize nitrite and slow its depletion, consistent with previous observation in vegetable-based curing systems [59,60]. This interaction underscores the role of ascorbic acid as a critical reductant that facilitates the conversion of nitrite to nitric oxide, thereby lowering residual nitrite levels, particularly in plant-based curing systems [23,58,60]. Therefore, when employing plant-derived nitrite sources such as PCCP, it is essential to consider the accompanying nitrate content and apply appropriate reducing agents to ensure effective control of residual nitrite in cured meat products.

## 4. Conclusions

This study demonstrated that CEP, when combined with PCCP, can serve as an effective natural curing accelerator in clean-label pork sausages. CEP significantly enhanced nitrosyl hemochrome formation, total pigment retention, and curing efficiency, particularly at high concentrations. Although instrumental redness (CIE a*) decreased slightly at high CEP levels, this did not correspond to a reduction in cured meat pigment, indicating that the intrinsic colorants in plant-based extracts may mask redness without impairing pigment formation. Sodium ascorbate markedly improved curing efficiency and reduced residual nitrite levels, whereas PCCP exhibited nitrite functionality comparable to that of synthetic sodium nitrite. These results support the feasibility of replacing synthetic curing agents with CEP and PCCP in naturally cured meat products. Nevertheless, this study is limited in that it focused mainly on physicochemical parameters and did not assess sensory attributes or microbiological safety. Future research should address these aspects to fully validate the applicability of PCCP and CEP in commercial clean-label meat processing.

## Figures and Tables

**Table 1:** Experimental design (3 × 2 × 2 factorial) for evaluating the effects of clove extract powder, sodium ascorbate, and nitrite source on physicochemical characteristics of cured pork sausages.

No.	Clove Extract Powder ^1^	Sodium Ascorbate ^2^	Nitrite Source ^3^
1	0 ppm	0 ppm	Synthetic sodium nitrite
2	0 ppm	0 ppm	Pre-converted Chinese cabbage powder
3	0 ppm	500 ppm	Synthetic sodium nitrite
4	0 ppm	500 ppm	Pre-converted Chinese cabbage powder
5	500 ppm	0 ppm	Synthetic sodium nitrite
6	500 ppm	0 ppm	Pre-converted Chinese cabbage powder
7	500 ppm	500 ppm	Synthetic sodium nitrite
8	500 ppm	500 ppm	Pre-converted Chinese cabbage powder
9	1000 ppm	0 ppm	Synthetic sodium nitrite
10	1000 ppm	0 ppm	Pre-converted Chinese cabbage powder
11	1000 ppm	500 ppm	Synthetic sodium nitrite
12	1000 ppm	500 ppm	Pre-converted Chinese cabbage powder

^1^ Clove extract powder: Samples were formulated with 0, 500, or 1000 ppm clove extract powder. ^2^ Sodium ascorbate: Samples were prepared with or without 500 ppm sodium ascorbate. ^3^ Nitrite source: Two nitrite sources (0.01% sodium nitrite or 0.44% pre-converted Chinese cabbage powder) were used to provide an equivalent nitrite content.

**Table 2:** Significance of main and interaction effects of clove extract powder, sodium ascorbate, and nitrite source on the physicochemical characteristics of naturally cured pork sausages.

Main and Interaction Effects	pH	Cooking Loss	CIE L*	CIE a*	CIE b*	Residual Nitrite	Nitrosyl Hemochrome	Total Pigment	Curing Efficiency
Clove Extract Powder (C) ^1^	<0.01	<0.01	<0.01	<0.01	<0.01	<0.01	<0.01	<0.01	<0.05
Sodium Ascorbate (S) ^2^	NS	NS	NS	NS	<0.01	<0.01	<0.01	NS	<0.01
Nitrite Source (N) ^3^	NS	<0.01	NS	NS	NS	<0.01	NS	NS	NS
C × S	NS	NS	NS	NS	<0.05	NS	NS	<0.05	NS
C × N	NS	NS	NS	NS	NS	NS	NS	NS	NS
S × N	NS	NS	NS	NS	NS	<0.01	NS	NS	NS
C × S × N	NS	NS	NS	NS	NS	NS	NS	NS	NS

Main and interaction effects: *p*-values are presented as significance levels (<0.05, <0.01) or as NS (no significant difference, *p* ≥ 0.05). Full *p*-values are provided in Supplementary Table S2. ^1^ Clove extract powder: Samples were formulated with 0, 500, or 1000 ppm clove extract powder. ^2^ Sodium ascorbate: Samples were prepared without or with 500 ppm sodium ascorbate. ^3^ Nitrite source: Two nitrite sources (0.01% sodium nitrite or 0.44% pre-converted Chinese cabbage powder) were used to provide an equivalent nitrite content.

**Table 3:** Effects of clove extract powder, sodium ascorbate, and nitrite source on pH, cooking loss, and CIE color of cured pork sausages.

Main Effects	pH	Cooking Loss (%)	CIE L*	CIE a*	CIE b*
Clove Extract Powder ^1^					
0 ppm	6.13 ^A^	4.72 ^B^	66.81 ^A^	11.35 ^A^	7.26 ^C^
500 ppm	6.12 ^A^	5.38 ^B^	64.49 ^B^	11.27 ^AB^	8.42 ^B^
1000 ppm	6.09 ^B^	6.77 ^A^	63.47 ^C^	11.17 ^B^	10.50 ^A^
SEM	0.02	0.74	0.35	0.10	0.14
Sodium Ascorbate ^2^					
Non-Added	6.11 ^A^	5.60 ^A^	64.86 ^A^	11.26 ^A^	8.48 ^B^
Added	6.12 ^A^	5.65 ^A^	64.98 ^A^	11.27 ^A^	8.96 ^A^
SEM	0.02	0.72	0.34	0.09	0.12
Nitrite Source ^3^					
Sodium Nitrite	6.11 ^A^	6.08 ^A^	65.04 ^A^	11.25 ^A^	8.68 ^A^
PCCP	6.12 ^A^	5.16 ^B^	64.80 ^A^	11.28 ^A^	8.76 ^A^
SEM	0.02	0.72	0.34	0.09	0.12

^1^ Clove extract powder: Samples were formulated with 0, 500, or 1000 ppm clove extract powder. ^2^ Sodium ascorbate: Samples were prepared without or with 500 ppm sodium ascorbate. ^3^ Nitrite source: Two nitrite sources (0.01% sodium nitrite or 0.44% pre-converted Chinese cabbage powder) were used to provide an equivalent nitrite content. ^A, B, C^ Different superscript letters within a column indicate significant differences (*p* < 0.05).

**Table 4:** Effects of clove extract powder, sodium ascorbate, and nitrite source on residual nitrite, nitrosyl hemochrome, total pigment, and curing efficiency of cured pork sausages.

Main Effects	Residual Nitrite (ppm)	Nitrosyl Hemochrome (ppm)	Total Pigment (ppm)	Curing Efficiency (%)
Clove Extract Powder ^1^				
0 ppm	36.36 ^A^	36.37 ^B^	48.27 ^B^	75.53 ^B^
500 ppm	34.52 ^B^	38.24 ^B^	48.85 ^B^	78.30 ^A^
1000 ppm	33.41 ^C^	40.30 ^A^	50.46 ^A^	79.95 ^A^
SEM	1.39	1.23	1.06	2.66
Sodium Ascorbate ^2^				
Non-Added	54.10 ^A^	36.33 ^B^	49.14 ^A^	73.99 ^B^
Added	15.42 ^B^	40.27 ^A^	49.24 ^A^	81.86 ^A^
SEM	1.37	1.17	1.05	2.52
Nitrite Sources ^3^				
Sodium Nitrite	32.08 ^B^	38.02 ^A^	49.33 ^A^	77.09 ^A^
PCCP	37.45 ^A^	38.59 ^A^	49.05 ^A^	78.76 ^A^
SEM	1.37	1.17	1.05	2.52

^1^ Clove extract powder: Samples were formulated with 0, 500, or 1000 ppm clove extract powder. ^2^ Sodium ascorbate: Samples were prepared without or with 500 ppm sodium ascorbate. ^3^ Nitrite source: Two nitrite sources (0.01% sodium nitrite or 0.44% pre-converted Chinese cabbage powder) were used to provide an equivalent nitrite content. ^A, B, C^ Different superscript letters within a column indicate significant differences (*p* < 0.05).

**Table 5:** Interaction effects of sodium ascorbate with clove extract powder or nitrite source on CIE b*, total pigment, and residual nitrite in cured pork sausages.

Dependent Variables	Interaction Effects	Sodium Ascorbate ^1^	
		Non-Added	Added
CIE b*	Clove Extract Powder ^2^		
	0 ppm	7.23 ^Cx^	7.29 ^Cx^
	500 ppm	7.90 ^By^	8.93 ^Bx^
	1000 ppm	10.33 ^Ax^	10.67 ^Ax^
Total Pigment (ppm)	Clove Extract Powder ^2^		
	0 ppm	48.82 ^Bx^	47.71 ^Cy^
	500 ppm	48.34 ^Bx^	49.36 ^Bx^
	1000 ppm	50.26 ^Ax^	50.66 ^Ax^
Residual Nitrite (ppm)	Nitrite Source ^3^		
	Sodium Nitrite	50.49 ^Bx^	13.67 ^By^
	PCCP	57.71 ^Ax^	17.18 ^Ay^

^1^ Sodium ascorbate: Samples were prepared without or with 500 ppm sodium ascorbate. ^2^ Clove extract powder: Samples were formulated with 0, 500, or 1000 ppm clove extract powder. ^3^ Nitrite source: Two nitrite sources (0.01% sodium nitrite or 0.44% pre-converted Chinese cabbage powder) were used to provide an equivalent nitrite content. ^A, B, C^ Different superscript letters within a column indicate significant differences (*p* < 0.05). ^x, y^ Different superscript letters within a row indicate significant differences (*p* < 0.05).

## Data Availability

The original contributions presented in the study are included in the article/Supplementary Materials, further inquiries can be directed to the corresponding author.

## Supplementary Materials

The following supporting information can be downloaded at https://www.mdpi.com/article/10.3390/foods14193316/s1, Table S1. Formulations of cured pork sausages prepared with clove extract powder, sodium ascorbate, and nitrite source; Table S2. Significance of main and interaction effects of clove extract powder, sodium ascorbate, and nitrite source on the physicochemical characteristics of cured pork sausages.

## Abbreviations

The following abbreviations are used in this manuscript:

CEP	Clove extract powder
PCCP	Pre-converted Chinese cabbage powder
TPC	Total phenolic content
GAE	Gallic acid equivalents
DPPH	2,2-diphenyl-1-picrylhydrazyl
TE	Trolox equivalents
