# Supplementary material for: Chinese Cabbage Powder and Clove Extract as Natural Alternatives to Synthetic Nitrite and Ascorbate in Clean-Label Pork Sausages
Source: Foods. 2025 Sep 24;14(19):3316. doi: 10.3390/foods14193316 (PMC12523696; doi:10.3390/foods14193316)
Supplement: Supplementary file 1 [file foods-14-03316-s001.zip › foods-3868357-supplementary/Table S1..pdf]

Table S1. Formulations of cured pork sausages prepared with clove extract powder, sodium ascorbate, and nitrite source.

Materials and Ingredients (%)	Sample No.											
	1	2	3	4	5	6	7	8	9	10	11	12
Pork ham	70.00	70.00	70.00	70.00	70.00	70.00	70.00	70.00	70.00	70.00	70.00	70.00
Pork back fat	15.00	15.00	15.00	15.00	15.00	15.00	15.00	15.00	15.00	15.00	15.00	15.00
Ice/water	15.00	15.00	15.00	15.00	15.00	15.00	15.00	15.00	15.00	15.00	15.00	15.00
Sub-total	100.00	100.00	100.00	100.00	100.00	100.00	100.00	100.00	100.00	100.00	100.00	100.00
Sodium chloride	2.00	2.00	2.00	2.00	2.00	2.00	2.00	2.00	2.00	2.00	2.00	2.00
Dextrose	1.00	1.00	1.00	1.00	1.00	1.00	1.00	1.00	1.00	1.00	1.00	1.00
Sodium nitrite	0.01	0.00	0.01	0.00	0.01	0.00	0.01	0.00	0.01	0.00	0.01	0.00
Pre-converted Chinese cabbage powder	0.00	0.44	0.00	0.44	0.00	0.44	0.00	0.44	0.00	0.44	0.00	0.44
Sodium ascorbate	0.00	0.00	0.05	0.05	0.00	0.00	0.05	0.05	0.00	0.00	0.05	0.05
Clove extract powder	0.00	0.00	0.00	0.00	0.05	0.05	0.05	0.05	0.10	0.10	0.10	0.10
Total	103.01	103.44	103.06	103.49	103.06	103.49	103.11	103.54	103.11	103.54	103.16	103.59
