# Supplementary material for: Chinese Cabbage Powder and Clove Extract as Natural Alternatives to Synthetic Nitrite and Ascorbate in Clean-Label Pork Sausages
Source: Foods. 2025 Sep 24;14(19):3316. doi: 10.3390/foods14193316 (PMC12523696; doi:10.3390/foods14193316)
Supplement: Supplementary file 1 [file foods-14-03316-s001.zip › foods-3868357-supplementary/Table S2..pdf]

Table S2. *p*-values for of main and interaction effects of clove extract powder, sodium ascorbate, and nitrite source on the physicochemical characteristics of cured pork sausages.

Main and Interaction Effects	pH	Cooking Loss	CIE L*	CIE a*	CIE b*	Residual Nitrite	Nitrosyl Hemochrome	Total Pigment	Curing Efficiency
Clove Extract Powder (C)[1]	< 0.01	< 0.01	< 0.01	< 0.01	< 0.01	< 0.01	< 0.01	< 0.01	0.03
Sodium Ascorbate (S)[2]	0.49	0.87	0.40	0.80	< 0.01	< 0.01	< 0.01	0.73	< 0.01
Nitrite Source (N)[3]	0.13	< 0.01	0.12	0.35	0.57	< 0.01	0.45	0.36	0.35
C × S	0.96	0.17	0.28	0.71	0.03	0.13	0.89	0.02	0.51
C × N	0.90	0.62	0.76	0.22	0.93	0.76	0.96	0.32	0.81
S × N	0.59	0.84	0.53	0.21	0.76	< 0.01	0.54	0.51	0.48
C × S × N	0.64	0.91	0.51	0.73	1.00	0.44	0.82	0.83	0.94

[1] Clove extract powder: Samples were formulated with 0, 500, or 1000 ppm clove extract powder.

[2] Sodium ascorbate: Samples were prepared with or without 500 ppm sodium ascorbate.

[3] Nitrite source: Two nitrite sources (0.01% sodium nitrite or 0.44% pre-converted Chinese cabbage powder) were used to provide an equivalent nitrite content.
